# *Aggregatibacter actinomycetemcomitans* and *Filifactor alocis* as Associated with Periodontal Attachment Loss in a Cohort of Ghanaian Adolescents

**DOI:** 10.3390/microorganisms10122511

**Published:** 2022-12-19

**Authors:** Zeinab Razooqi, Carola Höglund Åberg, Francis Kwamin, Rolf Claesson, Dorte Haubek, Jan Oscarsson, Anders Johansson

**Affiliations:** 1 Department of Odontology, Umeå University, 901 87 Umeå, Sweden; 2 Dental School University of Ghana, Korle-Bu, Accra KB 460, Ghana; 3 Jammerbugt Municipal Dental Service, Skolevej 1, DK-9460 Brovst, Denmark

**Keywords:** periodontitis, adolescents, *Aggregatibacter actinomycetemcomitans*, *Filifactor alocis*, RTX proteins

## Abstract

The aims of the present study were to document the presence of *Aggregatibacter actinomyctemcomitans* and the emerging oral pathogen *Filifactor alocis*, as well as to identify genotypes of these bacterial species with enhanced virulence. In addition, these data were analyzed in relation to periodontal pocket depth (PPD) and the progression of PPD from the sampled periodontal sites during a two-year period. Subgingival plaque samples were collected from 172 periodontal pockets of 68 Ghanaian adolescents. PPD at sampling varied from 3–14 mm and the progression from baseline, i.e., two years earlier up to 8 mm. The levels of *A. actinomycetemcomitans* and *F. alocis* were determined with quantitative PCR. The highly leukotoxic JP2-genotype of *A. actinomycetemcomitans* and the *ftxA* a gene of *F. alocis*, encoding a putative Repeats-in-Toxin (RTX) protein, were detected with conventional PCR. The prevalence of *A. actinomycetemcomitans* was 57%, and 14% of the samples contained the JP2 genotype. *F. alocis* was detected in 92% of the samples and the *ftxA* gene in 52%. The levels of these bacterial species were significantly associated with enhanced PPD and progression, with a more pronounced impact in sites positive for the JP2 genotype or the *ftxA* gene. Taken together, the results indicate that the presence of both *A. actinomycetemcomitans* and *F. alocis* with their RTX proteins are linked to increased PPD and progression of disease.

## 1. Introduction

Periodontitis is a chronic, infection-induced, inflammatory disease that degrades the tooth-supporting tissues, bone and connective tissue [1,2]. It affects mostly middle-aged and elderly individuals, but could in exceptional cases also affect adolescents [3,4,5,6]. This latter rapidly progressing form of periodontitis is classified as grade C [7], and when it affects adolescents, it was previously diagnosed as aggressive periodontitis [8]. The global prevalence of this disease indicates a wide geographical spread [9]. High incidence of this disease can be found in adolescents from the northern and western part of Africa [10,11]. Longitudinal studies on adolescents in these geographic regions indicate a substantial role of the microbiota with high prevalence of a highly leukotoxic variant of the facultative anaerobic, Gram-negative bacterium *Aggregatibacter actinomycetemcomitans*, namely, the JP2 genotype [12,13,14]. Reports about bacterial species other than *A. actinomycetemcomitans* in relation to periodontitis in young individuals in these geographic regions are scarce [10,15].

The present cohort of Ghanaian school children has previously been longitudinally examined for the presence and progression of periodontal attachment loss [13,16]. The prevalence of *A. actinomycetemccomitas* was detected in 56% of individuals when analyzed by cultivation and conventional PCR in pooled subgingival plaque samples [16]. Presence of the highly leukotoxic JP2 genotype was detected in 8.8% of individuals in the studied population [16]. The JP2 genotype of this bacterium has a 530-base pair deletion in the promoter region of the operon encoding the RTX protein LtxA (Leukotoxin), a toxin which is expressed at high levels by this genotype [17]. Carriage of *A. actinomycetemcomitans* is associated significantly with periodontal attachment loss at base line, without any additional effect in individuals where the JP2 genotype could be detected [16]. Interestingly, the clinical examination at the two-year follow-up examination showed a substantially increased odds ratio (OR = 14.3) for disease progression in the JP2 carriers compared to the individuals with no detectable *A. actinomycetemcomitans* [13]. The corresponding OR for the carriers of the non-JP2 genotype of *A. actinomycetemcomitans* was significantly lower (OR = 3.4). These associations with disease progression are in line with a previous longitudinal study in a population of Moroccan adolescents [12]. The association between *A. actinomycetemcomitans* and disease progression has also been studied in adolescents in the United States (US), with a similar association between carriership of this bacterium and diseases progression [18]. In the same (US) study, however. the pronounced correlation of the JP2 genotype to disease progression could not be shown, as the carriers of this genotype were few. In a later report on this US population, in sites prior to bone loss, *A. actinomyctemcomitans* was present in a consortium with *Streptococcus parasanguinis*, and the anaerobic, Gram-positive bacterium *Filifactor alocis* [19]. Data from a South Indian population showed increased levels of *F. alocis* in subgingival plaque from chronic periodontitis patients, whereas levels of this species were lower in samples from the aggressive form [20]. A recent study showed that *F. alocis* also carries a gene encoding an RTX protein, which was referred to as *ftxA* [21]. The function and potential virulence of the encoded protein FtxA is still unknown, but its expression has been confirmed in proteomic characterization of several isolates of this bacterium [22]. We hypothesize that *A. actinomycetemcomitans* initiates periodontal attachment loss, which promotes a beneficial environment for proliferation of anaerobic oral bacterial species such as *F. alocis* [23]. In addition, we hypothesize that the expression of the two RTX proteins (LtxA and FtxA) is of significant importance. The aims of this study were to investigate the prevalence and levels of *A. actinomyecetemcomitans* and *F. alocis* in relation to the severity and/or progression of periodontal attachment loss. In addition, this study aimed to determine the relative roles of their JP2 and FtxA-positive genotypes, respectively, in periodontal attachment loss and progression.

## 2. Materials and Methods

### 2.1. Study Population

The study population consisted of 68 individuals with PPD ≥ 3 mm selected from a cohort of 500 longitudinally examined Ghanaian adolescents [13]. The mean age of the 68 individuals was 14.0 (±1.4) years at the follow-up (FU) sampling and 12.0 (±1.4) years at the baseline examination two years before. This study population consisted of generally healthy school children, examined at their first and third years at primary high schools in the Korle-Bu area of Accra, Ghana. Earlier reports have delivered a more detailed description of this population [16].

### 2.2. Clinical Assessments

The follow-up examination was conducted in November 2011 and included periodontal recordings of 397 (79.4%) of the 500 subjects originally included in the baseline study [13,16]. All clinical parameters were recorded by the same periodontist (CHÅ) at both the baseline (BL) and at the FU examination. The periodontal examination at the follow-up was performed according to the same procedure as at baseline and included the measurement of probing pocket depth (PPD) and clinical attachment loss (CAL). Samples collected from sites with PPD ≥ 3 mm among the 172 initial samples collected from the 68 individuals were included in this study. Three millimeters is a value that is commonly used as threshold to separate diseased sites from healthy sites [12]. Progression of PPD was calculated as the difference between the BL PPD with the two-year FU PPD for each of the 172 sites.

### 2.3. Sampling

**Figure 1:**
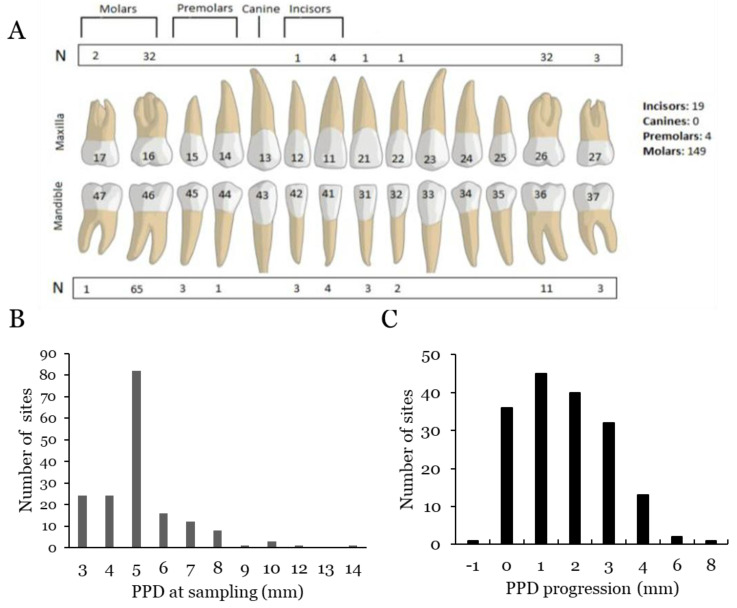
Illustration of number (N) and location of sampled sites (**A**), PPD at FU sampling (**B**), and progression of PPD (**C**) in the sampled sites from BL to FU examinations. Subgingival plaque samples are collected with paper points in the area between the teeth and the periodontal tissue.

Subgingival plaque samples from the 172 periodontal sites were collected for microbiological analysis, as described earlier [16]. Briefly, subgingival plaque samples were collected from the selected sites (PPD ≥ 3 mm) by paper points for 30 s, which were subsequently transferred to a tube containing 2 mL viability-preserving, microbiostatic, anaerobic media (VMGAIII) [24]. All 172 samples were collected during the two-year FU examination. A courier was used for transportation of the samples, sent from Accra, Ghana, to the Dental School, Odontology, Umeå University. Immediately after arrival, aliquots of the samples were spread on blood agar and *A. actinomycetemcomitans*-selective agar plates, respectively [16]. The remaining amounts of the samples were frozen at −80 °C prior to the present analysis. The distributions of sampled sites, PPD, and progression are shown in Figure 1.

### 2.4. DNA Isolation

The GXT NA extraction kit (DiaSorin Ltd., Dublin, Ireland) was used for DNA isolation, and for the procedure, an automated extraction instrument was used (Liaison IXT, DiaSorin Ltd., Ireland). We mixed 200 µL of the samples with 600 µL 1M Tris buffer (pH 8.0), and DNA was extracted from 550 μL of this sample mixture and eluted in a volume of 100 μL. Suspensions of reference strains *A. actinomyceemcomitans* (HK1651) [25] and *F. alocis* (ATCC 35896) [26,27] (10^9^ cells/mL) were treated as described above and used for standard curves and hence serially diluted at different concentrations. The samples and the standard solutions were stored at 4 °C until use.

### 2.5. Microbiological Analyses

#### 2.5.1. Quantitative PCR

Quantitative PCR was used to determine levels of *A. actinomycetemcomitans* and *F. alocis* cells in the samples [28,29]. Isolated DNA from samples and the standards solution (10^8^–10^1^ cells/mL) were analyzed in duplicates by using a Corbett Research Rotor-Gene 6000 Rotary Analyze instrument (QIAGEN, Valencia, CA, USA).

Quantification of the total concentration of the two bacterial species in the samples was performed according to Kirakodu and co-workers [28] and Siqueira and Rôças [29], respectively. The species-specific oligonucleotide primers and the PCR cycling conditions used for these two quantification methods are indicated in Table S1.

#### 2.5.2. Conventional PCR

For detection of the JP2 genotype, specific oligonucleotide primers targeting the leukotoxin promoter sequence of *A. actinomycetemcomians* were used [30]. Presence of the *ftxA* gene of *F. alocis* in the samples was detected according to Oscarsson and co-workers [21]. The species-specific primers and the PCR programs for these two detections methods are shown in Table S2.

### 2.6. Statistical Analyses

Data analyses were performed using SPSS 22.0 (SPSS Inc., Chicago, IL, USA). In the statistical analyses, the primary outcome was the PPD or progression associated to bacterial load of the sampled site. Significant differences between sample groups were examined with the Mann–Whitney U test or *t*-test.

### 2.7. Ethics Statement

Ethical approval for the study was obtained from the Noguchi Memorial Institute for Medical Research, University of Ghana (IRB 000 1276), and from the local Ethical committee of Umeå University, Sweden (Dnr 2010-188-31M). Signed consent was received from the parents or the guardians of the children before they entered the study.

## 3. Results

### 3.1. A. actinomycetemcomitans

#### 3.1.1. Prevalence and Levels of *A. ctinomycetemcomitans*

**Figure 2:**
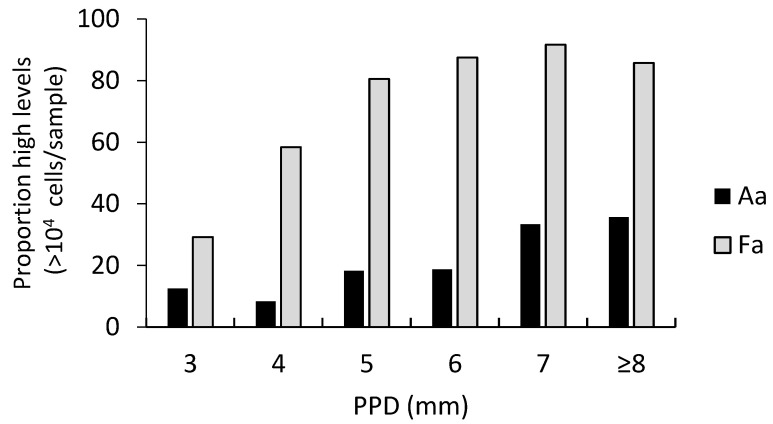
Proportion of samples with high levels (>10^4^ cells/sample) of *A. actinomycetemcomitans* (Aa) or *F. alocis* (Fa), respectively, in relation to PPD.

*A. actinomycetemcomitans* was detected in 55% (n = 95) of the sampled sites, and the highly leukotoxic JP2 genotype of this bacterium was detected in 14% (n = 24). The proportion of samples with higher levels of *A. actinomycetemcomitans* (>10^4^/sample) was 19% (n = 33). The mean level of this bacterium in the 95 positive samples was 15.4 × 10^4^ cells/sample (median 0.26 × 10^3^). The distribution of samples with high levels (>10^4^/sample) of *A. actinomycetemcomitans* in relation to PPD is shown in Figure 2.

**Figure 3:**
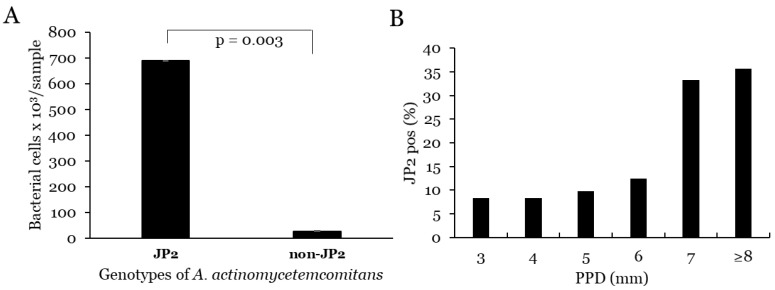
(**A**) Mean levels of *A. actinomycetemcomitans* cells as determined in sites with presence (JP2) and absence (non-JP2) of this genotype of the bacterium. (**B**) Proportion of JP2-positive sites associated with PPD.

In comparison, between *A. actinomycetemcomitans*-positive samples with and without the JP2 genotype, the levels of cells of this species were significantly higher in the JP2-positive samples (*p* = 0.003) (Figure 3).

#### 3.1.2. Correlation with PPD and Progression for *A. actinomycetemcomitans*

The levels of *A. actinomycetemcomitans* were not significantly correlated with increased PPD or with progression (Table 1). Samples with high levels of *A. actinomycetemcomitans* (>10,000 cells/sample) were significantly more prevalent from sites with increased PPD (*p* = 0.028) (Table 1) but were not associated with increased attachment loss progression (*p* = 0.076) (Table 1). The JP2-positive samples were significantly associated with sites with increased PPD (*p* = 0.024), as well as with progression (*p* = 0.003) (Table 1). The highest levels of PPD, as well as progression, were in sites positive for both the JP2 genotype and the *ftxA* gene of *F. alocis* (Table 1).

### 3.2. F. alocis

#### 3.2.1. Prevalence and Levels of *F. alocis*

**Figure 4:**
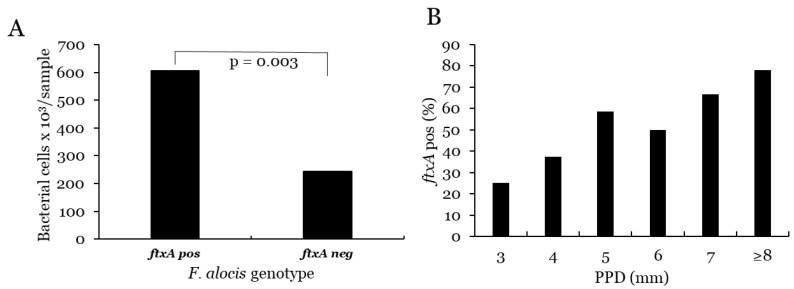
(**A**) Mean levels of *F. alocis* cells in sites positive for *this bacterium* with (*ftxA* pos) or without (*ftxA* neg) presence of the *ftxA* gene. (**B**) Proportion of *ftxA*-positive sites associated with PPD.

The prevalence of *F. alocis* was 92% (n = 157) of the sampled sites, and the *ftxA* gene of this bacterium was detected in 52% of samples (n = 90). The proportion of samples with higher levels of *F. alocis* (>10,000 cells/sample) was 72% (n = 123). The mean level of this bacterium in the 157 positive samples was 40.8 × 10^4^ cells/sample (median 10.5 × 10^4^). The distribution of samples with higher levels (>10^4^ cells/sample) of *F. alocis* in relation to PPD is shown in Figure 2. In comparison, between *F. alocis*-positive samples with and without presence of the *ftxA* gene, the levels of *F. alocis* were significantly higher in the *ftxA*-positive samples (*p* = 0.003) (Figure 4).

#### 3.2.2. Correlation with PPD and Progression for *F. alocis*

The prevalence of *F. alocis* was significantly associated with increased PPD (*p* = 0.015) but not with progression (*p* = 0.236). Samples with high levels of *F. alocis* (>10 000) were significantly more prevalent in samples from sites with increased PPD (*p* < 0.001) (Table 1), but not to increased progression (*p* < 0.001) (Table 1). The *ftxA*-positive samples were significantly associated with sites with increased PPD (*p* = 0.005), but not with progression (*p* = 0.123) (Table 1). In combination with JP2, the *ftxA*-positive sites showed the highest levels of PPD and progression (Table 1).

## 4. Discussion

Data from the present study confirmed the strong association between the presence of *A. actinomycetemcomitans* and progression of periodontal attachment loss [18,31]. In line with previous findings, the presence of the highly leukotoxic JP2 genotype of this bacterium further increased the correlation to onset and progression of periodontitis [12,13]. Earlier reported data are based of the prevalence on an individual basis, while the present study provided quantitative data from specific periodontal sites. It has previously been shown that the high levels of the JP2 genotype in saliva are associated to periodontal attachment loss [32]. A similar trend was found in the present study, where the sampled levels of *A. actinomycetemcomitans* in subgingival plaque were significantly increased between the two examinations in the sites positive for the JP2 genotype. 

The RTX protein LtxA, which is highly expressed by the JP2 genotype, efficiently kills leukocytes during a rapid activation of a pro-inflammatory host response [33]. These properties of LtxA at least partly explain the strong correlation between the carriage of the JP2 genotype of *A. actinomycetemcomitans* and the onset and progression of periodontal attachment loss [34,35].

It has previously been reported that presence of *F. alocis* enhances the pathogenicity of *A. actinomycetemcomitans* [19]. Interestingly, it was recently demonstrated that *F. alocis* also encodes and expresses an RTX protein, FtxA [21,22]. The role of this potential virulence factor in periodontitis, as well as its mechanisms of action in host interaction, remain to be discovered. In the present study, we found a significant correlation between sample levels of *F. alocis* cell numbers and PPD or progression at the sampled site. Interestingly, the highest levels of PPD, as well as attachment loss progression, were documented in sites positive for both the JP2 genotype of *A. actinomycetemcomitans* and for *ftxA*-positive *F. alocis*. This is the first report that indicates a potential role of FtxA in the virulence of *F. alocis*. However, the lack of data for total bacterial loads in the samples of the present study made calculations of proportions in the microflora impossible. 

It has recently been reported that the exposure of neutrophils to *F. alocis* expanded their lifespan by downregulating pro-apoptotic genes and upregulating anti-apoptotic genes [36]. Although *F. alocis* strains known to encode *ftxA* evidently express this protein, refs. [21,22] it is not known if this RTX protein is involved in the effects observed on neutrophils [36,37]. In contrast, LtxA is known to activate and efficiently kill all subsets of leukocytes [33]. In addition, LtxA activates bone resorption through promoting osteoclast differentiation by activating the NLRP3 inflammasome and, consequently, the release of bioactive IL-1β [38,39]. How the prolonged leukocyte survival induced by *F. alocis* interferes with the pro-inflammatory response by LtxA remains to be discovered [23,40]. Despite the gap in knowledge concerning mechanisms of action of FtxA and its possible interaction with LtxA, it may function as a potential risk marker, alone or in combination with virulent genotypes of *A. actinomycetemcomitans*. As the mode of action of FtxA, and its potential role in periodontal disease, is not yet understood, this aspect may be considered a limitation of the present study.

## 5. Conclusions

*A. actinomycetemcomitans* and *F. alocis* are common inhabitants in the biofilm associated with sites with periodontal attachment loss of Ghanaian adolescents. The levels of these two periodontitis-associated species were significantly associated with both enhanced PPD and tooth attachment loss progression. These associations were further pronounced, if there was a co-carriage of the highly leukotoxic JP2 genotype of *A. actinomycetemcomitans* and *ftxA*-positive *F. alocis*.

## Figures and Tables

**Table 1:** PPD and progression in relation to carriership of *A. actinomycetemcomitans* (Aa) and *F. alocis* (Fa) in the sampled sites. Significant differences in clinical parameters between sample groups were calculated with the Mann–Whitney U test.

		PPD				Progresssion		
	N	Mean (mm)	SD	*p*-Value	N	Mean (mm)	SD	*p*-Value
Aa pos	95	5.24	±1.43	0.027	95	1.83	±1.41	0.163
Aa neg	77	5.05	±1.91		75	1.57	±1.41	
Aa high	33	5.67	±1.80	0.027	33	2.03	±1.55	0.225
Aa low	139	5.04	±1.61		137	1.64	±1.37	
JP2	24	5.79	±1.58	0.013	23	2.48	±1.54	0.009
Non-JP2	148	5.05	±1.66		147	1.60	±1.36	
Fa pos	157	5.24	±1.68	0.016	155	1.74	±1.41	0.384
Fa neg	14	4.21	±1.12		14	1.43	±1.45	
Fa high	123	5.49	±1.69	0.001	121	1.93	±1.37	0.001
Fa low	48	4.31	±1.27		48	1.17	±1.39	
*ftxA* pos	90	5.52	±1.65	0.001	89	1.85	±1.60	0.398
*ftxA* neg	82	4.76	±1.59		81	1.57	±1.16	
ftxA-JP2	15	6.20	±1.32	0.001	15	2.93	±1.53	0.002
Other	157	5.06	±1.66		155	1.60	±1.35	

## Data Availability

Data are available from the corresponding author (A.J.).

## Supplementary Materials

The following supporting information can be downloaded at: https://www.mdpi.com/article/10.3390/microorganisms10122511/s1, Table S1: Primer sequences; Table S2: Cycle settings for PCR.
