# Correction: Razooqi et al. *Aggregatibacter actinomycetemcomitans* and *Filifactor alocis* as Associated with Periodontal Attachment Loss in a Cohort of Ghanaian Adolescents. *Microorganisms* 2022, *10*, 2511

**DOI:** 10.3390/microorganisms14020432

**Published:** 2026-02-12

**Authors:** Zeinab Razooqi, Carola Höglund Åberg, Francis Kwamin, Rolf Claesson, Dorte Haubek, Jan Oscarsson, Anders Johansson

**Affiliations:** 1 Department of Odontology, Umeå University, 901 87 Umeå, Sweden; 2 Dental School University of Ghana, Korle-Bu, Accra KB 460, Ghana; 3 Jammerbugt Municipal Dental Service, Skolevej 1, DK-9460 Brovst, Denmark

In the original publication [1], there was a mistake in Table S1, Primer sequences for Fa, forward and reversed, as published. A PDF file has been enclosed with the correction of these primer sequences. The authors state that the scientific conclusions are unaffected. This correction was approved by the Academic Editor. The original publication has also been updated.
