# Supplementary material for: Aggregatibacter actinomycetemcomitans and Filifactor alocis as Associated with Periodontal Attachment Loss in a Cohort of Ghanaian Adolescents
Source: Microorganisms. 2022 Dec 19;10(12):2511. doi: 10.3390/microorganisms10122511 (PMC9781193; doi:10.3390/microorganisms10122511)
Supplement: Supplementary file 1 [file microorganisms-10-02511-s001.zip › microorganisms-2096528-supplementary.pdf]

Table S1

a. Primers used for qPCR - based quantification of *Aggregatibacter actinomyetemcomitans* and *Filifactor alocis* in accordance to Kirakodu et al. [18] and Siquiera & Rocas [19].

b. Cycle settings for quantification of *A. actinomycetemcomitans* and *F. alocis* in accordance to Kirakodu et al. [18] and Siquiera & Rocas [19].

**a**	Forward	Reverse
Kirakodu<br>*Aa (ltxA)*	CTAGGTATTGCGAAACAATTTG	CCTGAAATTAAGCTGGTAATC
Siquiera and Rocas<br>*Fa*	AGGCAGCTTGCCATACTGCG	ACTGTTAGCAACTACCGATGT

**b**	Kirakodu (*Aa*)	Siquiera and Rocas (*Fa*)
Hold/time	95°/10 min	95°/2 min
Cycling/time	95°/10 s	95°/30 s
Cycling/time	55°/5 s	55°/1 min (72°/2 min)
Cycling/time	72/10 s	72°/2 min
Cycles	45	40

Table S2
a. Primers used for detection of the *ltx* promoter gene of *A. actinomycetemcomitans* and the *ftxA* gene of *F. alocis* in accordance to Poulsen et. al. [20] and Oscarsson et al. [15].
b. Cycle settings for detecton of the *ltx promoter gene* of *A. actinomycetemcomitans* and the *ftxA* gene of *F. alocis* in accordance to Poulsen et. al. [20] and Oscarsson et al. [15].

**a**	Forward	Reverse
Poulsen<br>*ltx- promoter*	GCCGACACCAAAGACAAAGTCT	GCCCATAACCAAGCCACATAC
Oscarsson<br>*ftxA*	GGCTCAGATACCTACTTCTTC	GAAGGCTATGATTTGATTGTTTCC

**b**	Poulsen (*ltx promoter*)	Oscarsson (*ftxA*)
Hold/time	94°/5 min	95°/1 min
Cycling/time	94°/1 min	95°/30 s
Cycling/time	60°/1 min	54°/30 s
Cycling/time	72°/2 min	72°/1 min
Cycling/time	72°/8 min	72°/7 min
Cycles	30	35
